# Supplementary material for: Meron-like topological spin defects in monolayer CrCl3
Source: Nat Commun. 2020 Sep 18;11:4724. doi: 10.1038/s41467-020-18573-8 (PMC7501285; doi:10.1038/s41467-020-18573-8)
Supplement: Supplementary file 1 — Supplementary Information [file 41467_2020_18573_MOESM1_ESM.pdf]

Supplementary Information

Meron-Like Topological Spin Defects in Monolayer $CrCl_3$

Lu et al.

Supplementary Table 1. The MCA of $CrCl_3$ calculated by different Hubbard U and Hund J.

	U=1.5				U=2.7				U=3.5			
	MAE	A	λ_1	J_1	MAE	A	λ_1	J_1	MAE	A	λ_1	J_1
J=0	-32	-5.7	-3.3	-1416	-33	-4.2	-2.9	-1756	-37	-2.7	-2.6	-1878
J=0.7	-33	-6.5	-2.1	-232	-34	-6.5	-1.9	-792	-33	-5.2	-1.9	-982
J=1.5	-24	-11	-2.2	1223	-25	-11	0.03	435	-24	-13.8	-0.89	108

The unit for Hubbard U and Hund J is eV. The energy and coupling strength units are μeV.

Supplementary Table 2. The extracted magnetic interaction strengths with and without h-BN substrate.

	MSA($3\mu_B$)	MCA	MAE	A	λ_1	J_1	λ_2	J_2
W/O BN	-54	20	-34	-0.007	-0.002	-0.79	0.0004	-0.071
With BN	-54	23	-31	-0.0044	-0.0018	-0.81	-0.0011	-0.066

The unit for anisotropy energies and coupling strengths are μeV and meV respectively.

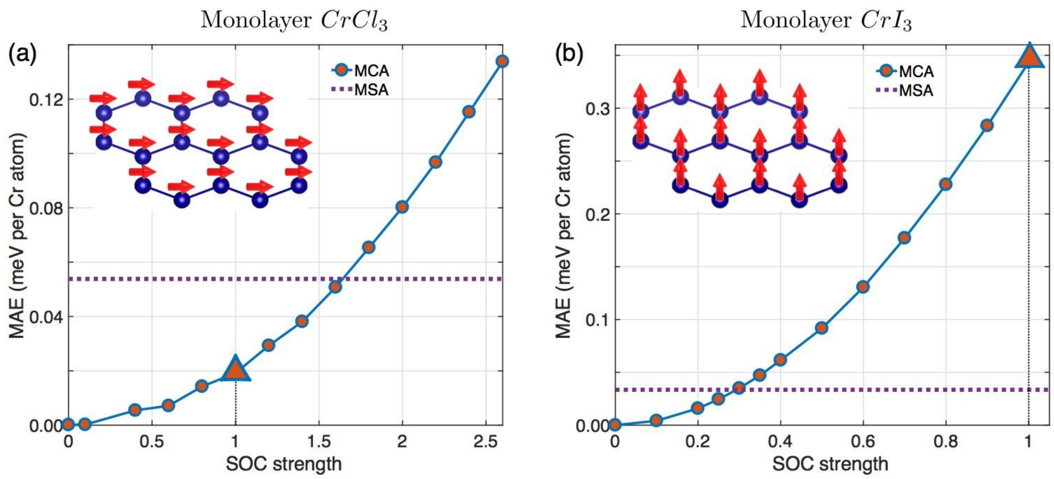

Supplementary Figure 1. Magnetic anisotropic energy of ML $CrCl_3$ and CrI_3. (a) and (b), the MCA energy as a function of SOC strength (strength=1 corresponds to the intrinsic material case) for ML $CrCl_3$ and ML CrI_3. The horizontal line is the absolute value of MSA energy (<0), which is induced by the D-D interaction. The insets are corresponding schematic directions of the magnetic polarization.

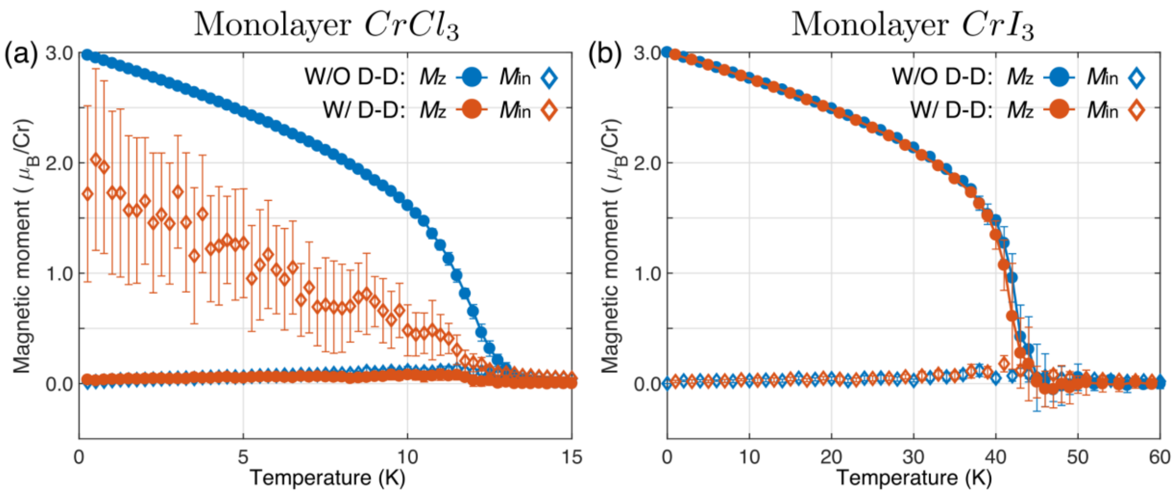

Supplementary Figure 2. The magnetization against temperature from MC simulations. (a), (b). The averaged magnetization of ML $CrCl_3$ and CrI_3 with (the red dotted line) and without (the blue dotted line) D-D interactions. The error bars are the standard deviation.

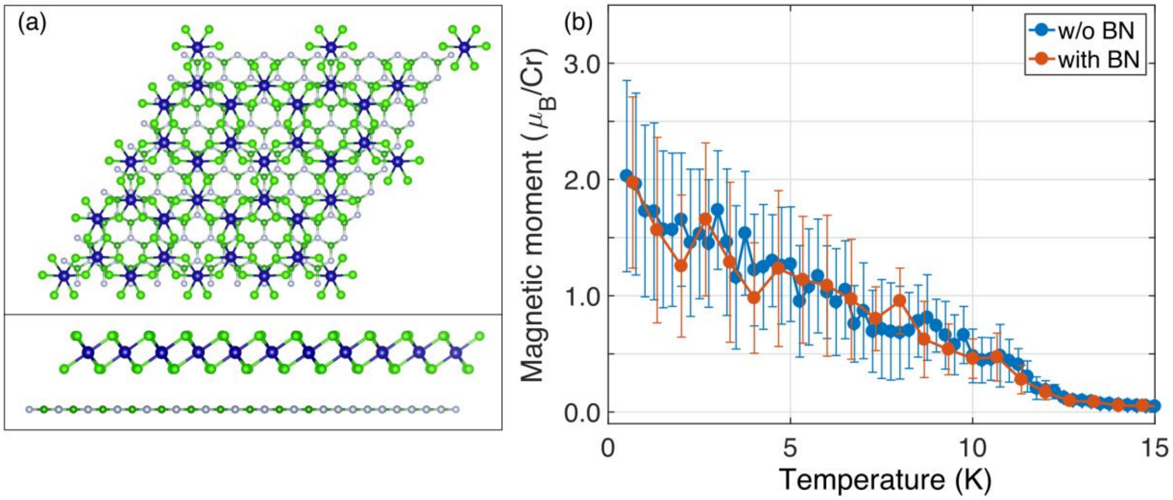

Supplementary Figure 3. Structure and magnetization for ML $CrCl_3$ attached with ML h-BN (a) Top and side views of the structure of ML $CrCl_3$ attached to ML h-BN. (b) The averaged magnetic moment with (red) and without (blue) the h-BN substrate. The error bars are the standard deviation.

Supplementary Figure 4. More examples of coupled topological defects pairs.

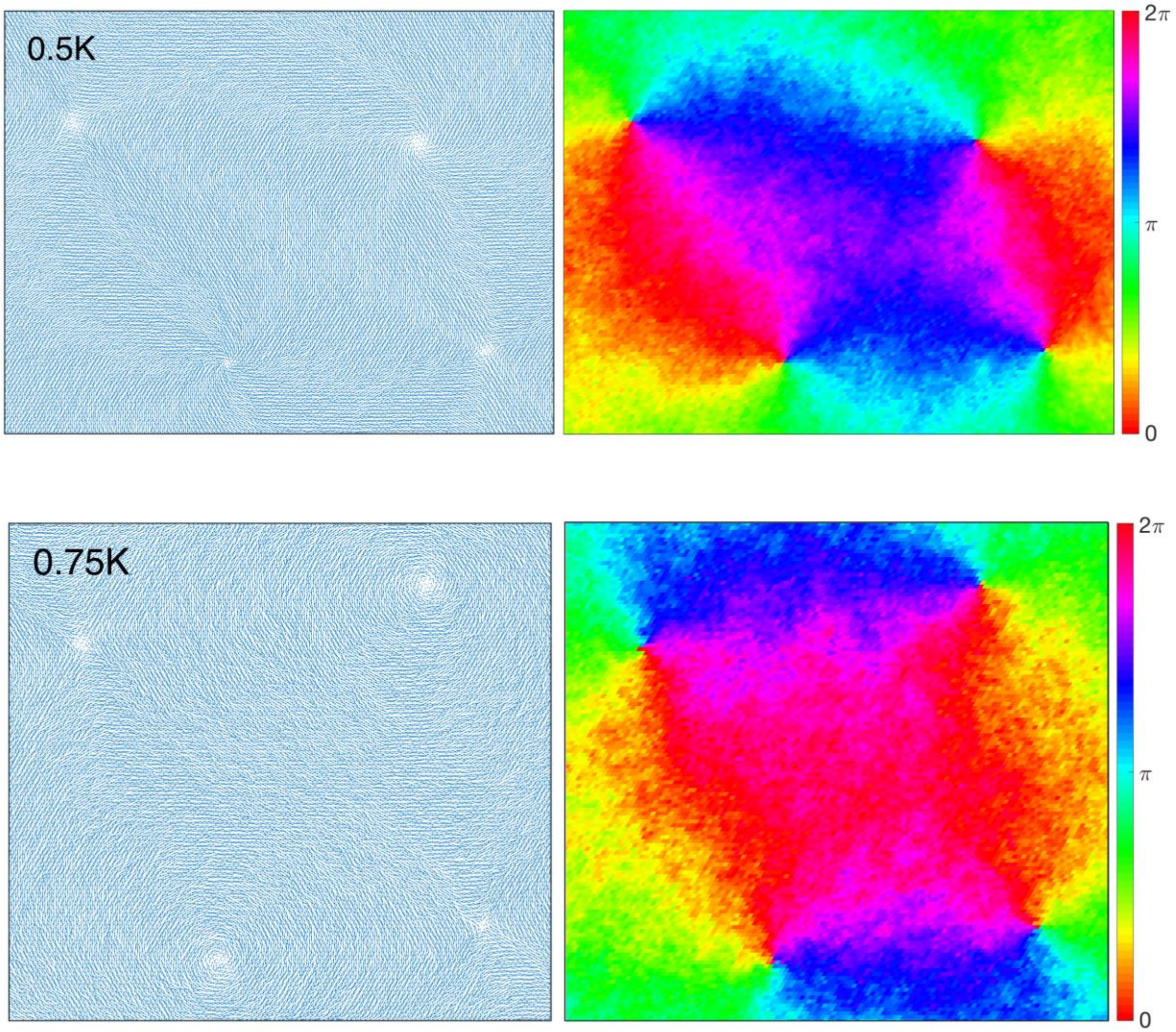

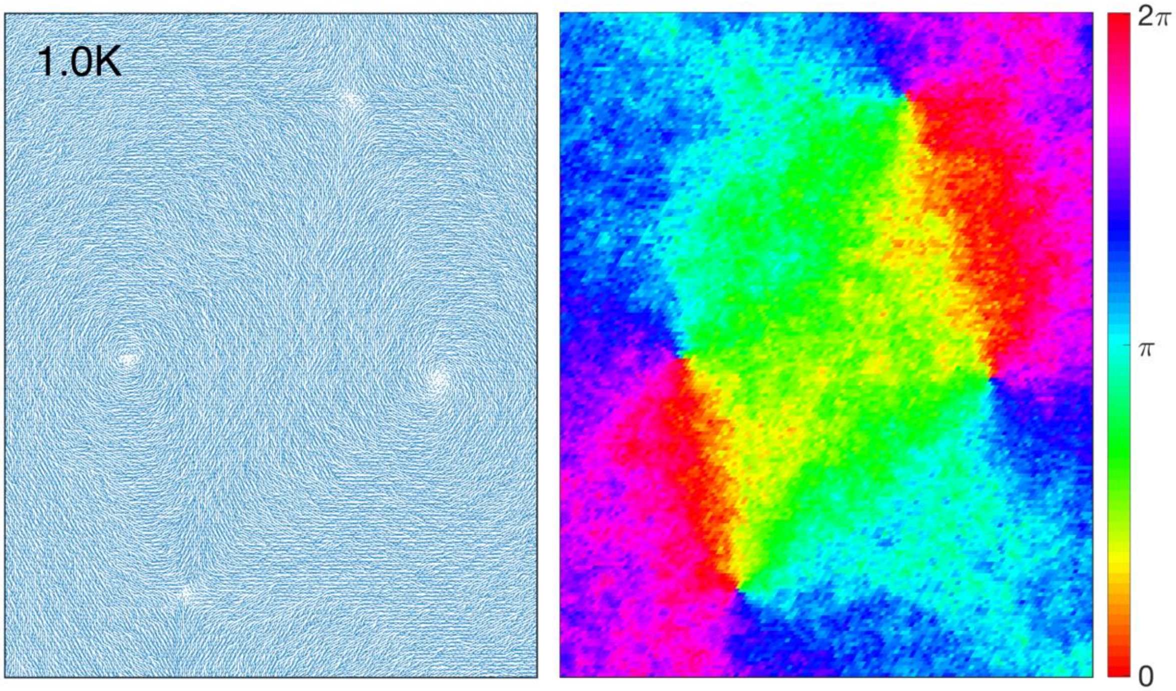

Right panel**:** top views of the real-space magnetic moments from snapshots of MC simulations under different temperatures. Left panel: phase map of in-plane magnetic moment component.

Supplementary Note 1. The impact of Hubbard U and Hund J on MCA energy.

The impact of Hubbard U and Hund J on MSA is summarized in Supplementary Table 1. We list the nearest couplings in Supplementary Table 1. From the table we can see that the magnetic anisotropy energy is not sensitive to the choice of U and J. Although large Hund J will increase MCA slightly, large Hund J makes the Heisenberg isotropic J_1 positive, which corresponds to AFM coupling. Therefore, large Hund J is not able to reproduce a correct ground magnetic state and is not a reasonable choice. With general choices of U and J, the MCA is always smaller than MSA, and shows little dependence on the values of U and J.

Supplementary note 2. The relation between spin-orbit coupling (SOC) strength and MCA energy.

As shown in Supplementary Figure 1, the MCA energy is proportional to SOC strength with roughly a quadratic relation ($\mathrm{MAE} \sim |\mathrm{SOC}|^2$). Through the comparison, we can see that the D-D interaction dominates the weak SOC effect in ML $CrCl_3$, resulting in an easy-plane anisotropy. However, in ML CrI_3, the strong SOC dominates the D-D interaction, and the easy-axis anisotropy renders an out-of-plane polarization.

Supplementary note 3: **The impact of D-D interactions on magnetization of ML $CrCl_3$ and CrI_3**

To show the crucial role of D-D interactions, we first turn it off. Consequently, ML $CrCl_3$ is dominated by the SOC induced easy-axis MCA. As shown in the Supplementary Figure 2 (a), it exhibits a significant out-of-plane magnetic polarization (the blue-dotted line M_z), and there is no in-plane magnetization (the blue diamond $M_{xy} \approx 0$). However, after including the D-D interaction (MSA) energy, we find that the out-of-plane magnetization (the blue-dotted line M_z) is almost completely quenched. The in-plane magnetization (the red diamond M_{in}) with large fluctuations emerges. In contrast to $CrCl_3$, ML CrI_3 exhibits a normal Ising-like magnetism. As shown in Supplementary Figure 2 (b), no matter with or without including the D-D interaction, the easy-axis MCA energy from strong SOC always dominates the overall MAE, rendering an out-of-plane magnetic ordering. The D-D interaction turns out to be negligible.

Supplementary note 4. Impact of substrate on monolayer $CrCl_3$

To ensure the feasibility of experimental observation of our predicted topological spin texture for monolayer $CrCl_3$, we also calculate the case where monolayer $CrCl_3$ is attached to a monolayer hexagonal boron nitride (h-BN), which is a common substrate used in material fabrications. To get commensurate heterostructure, the calculation is carried out through introducing 5x5x1 supercell h-BN to 2x2x1 $CrCl_3$ lattice with a -4% strain. The wide gap of h-BN

is not sensitive to the strain. The result is presented in Supplementary Figure 3. And Supplementary Table 2. There is no obvious difference of the average magnetic moment with (red) or without (blue) BN substrate. In conclusion, monolayer $CrCl_3$ attached to BN layer has similar behaviors compared with free-standing monolayer $CrCl_3$ except for the slightly modified magnetic interaction strengths. This shows the stability of meron states in proposed monolayer $CrCl_3$ against the substrate and provides a good chance for experimental observations since h-BN is widely used as an encapsulation material.
